# Tau Spreading Mechanisms; Implications for Dysfunctional Tauopathies

**DOI:** 10.3390/ijms19030645

**Published:** 2018-02-25

**Authors:** Almudena Fuster-Matanzo, Félix Hernández, Jesús Ávila

**Affiliations:** 1 Centro de Biología Molecular “Severo Ochoa” CSIC-UAM, Universidad Autónoma de Madrid, C/Nicolás Cabrera 1, 28049 Madrid, Spain; a.fuster@ms-c.es (A.F.-M.); fhernandez@cbm.csic.es (F.H.); 2 Centro de Investigación Biomédica en Red de Enfermedades Neurodegenerativas (CIBERNED), Valderrebollo 5, 28041 Madrid, Spain

**Keywords:** tau, tauopathies, spreading, prion-like transmission

## Abstract

Tauopathies comprise a group of progressive age-associated neurodegenerative diseases where tau protein deposits are found as the predominant pathological signature (primary tauopathies) or in combination with the presence of other toxic aggregates (secondary tauopathies). In recent years, emerging evidence suggests that abnormal tau accumulation is mediated through spreading of seeds of the protein from cell to cell, favouring the hypothesis of a prion-like transmission of tau to explain the propagation of the pathology. This would also support the concept that the pathology initiates in a very small part of the brain before becoming symptomatic and spreads across the brain over time. To date, many key questions still remain unclear, such as the nature of the tau species involved in the spreading, the precise seeding/template and uptaking mechanisms or the selectivity explaining why certain neurons are affected and some others are not. A better understanding of the tau spreading machinery will contribute to the development of new therapeutic approaches focused on halting the abnormal propagation, offering also new perspectives for early diagnosis and preventive therapies. In this review, we will cover the most recent advances in tau spreading mechanisms as well as the implications of these findings for dysfunctional tauopathies.

## 1. Introduction

Tuaopathies are a group of heterogeneous dementias with diverse phenotypic manifestations but with a common feature; intracellular accumulations of abnormal filaments formed by the microtubule-associated protein (MAP) tau [1]. Tauopathies are often difficult to diagnose antemortem and can be divided into two different categories: primary and secondary tauopathies (see Table 1). The first ones comprise disorders in which tau pathology is the major neuropathological characteristic, such as frontotemporal dementia with parkinsonism linked to chromosome 17 (FTDP-17) and Pick’s Disease. In the second ones, an additional driving force contributing to the disease is found, often another amyloid protein (β-amyloid peptide, Aβ, prion protein, PrP, 34-mer amyloid Bri, ABri). This group includes familial and sporadic Alzheimer’s Disease (AD), familial Gerstmann-Sträussler–Scheinker disease and familial British dementia [2]. Tauopathies can be also subclassified attending to the preferential accumulation of 3R or 4R tau [3], two different tau species resulting from the alternative splicing of exon 10. Thus, while in primary tauopathies there is a preferential accumulation of 3R, 4R or 3R + 4R tau depending on the disorder [4], in secondary tauopathies, including AD, tau is composed of and equimolar ratio of 3R and 4R tau (3R + 4R tau) [5] (Table 1).

Mounting evidence from animal and cell models suggests that pathogenic tau propagation between brain cells following a prion-like spreading mechanism is central to the neurodegenerative process. This concept was first introduced in 2009, when two different groups demonstrated that extracellular tau was able to propagate both in vitro [6] and in vivo [7]. Those experiments opened up a new pathway that keeps on being explored nowadays. Despite many questions that must be solved in the field, it has been suggested that therapeutics should focus on preventing the cellular release and uptake of the misfolded tau [8]. Understanding the underlying mechanisms of abnormal tau cell-to-cell transmission could provide, not only novel insights into the etiology of pathogenesis but could also help to identify new targets for the development of therapies focused on counteracting neurodegeneration or even preventing it. 

## 2. Tau Protein

Tau was first discovered in 1975 as a MAP, being the most abundant one in the brain [9]. The human tau gene (*MAPT*), located on chromosome 17q21, contains 16 exons, from which different tau isoforms are generated by alternative splicing [10,11]. Some of these isoforms are selectively expressed during embryonic and early postnatal development [11,12,13], whereas in the adult central nervous system, six different tau isoforms are expressed, differing in the presence or absence of exons 2, 3 and 10. Exon 10 encodes one of the four repeat sequences [5,14] that form the microtubule-binding domain [1]. The presence of exon 10 results in tau with four repeat microtubule-binding sequences (4R), whereas the alternatively spliced isoforms without exon 10 have only three of these sequences (3R). The expression of some of these tau isoforms is developmentally regulated. Thus, isoforms lacking exon 10 (3R) are found at early developmental stages whereas tau isoforms containing exon 10 (Tau 4R) are mainly found in neurons at mature developmental stages [15].

In the brain, tau is mainly found in neurons but it is also present at low levels in glia [16], and, despite being mostly an intracellular protein, it has also been detected outside cells as it will be further discussed later. Within neurons, tau is predominantly localized in the cytosol associated to microtubules, especially in the axonal compartment [17]. However, when phosphorylated, it can be also found in the somato-dendritic compartment [18] and even at dendritic spines [19]. Additionally, tau can associate with the plasma membrane [20,21] or it can be found in the nucleus [22]. Finally, tau expression in the human brain shows considerable regional variation. Protein levels in the white matter and the cerebellum are two-fold lower than the levels found in the neocortex. Importantly *MAPT* gene also exhibits regional differences [23,24]. All these variations in tau expression may contribute to the differential vulnerability of brain regions to tau pathology.

As stated, tau was originally discovered as a microtubule-associated protein, however, in the recent years, several other functions have been revealed. In neurons, besides regulating microtubule dynamics, tau may regulate axonal transport via different mechanisms, including its influence over the motor proteins dynein and kinesin with which tau competes for binding to microtubules, slowing down the anterograde and retrograde transport along the tubulin network [25]

However, despite this having been demonstrated in vitro, little influence of tau in axonal transport in vivo has been observed so far [26]. Also, in the axonal compartment, tau seems to be essential for axonal elongation and maturation as demonstrated in overexpression and knocking down experiments in vitro [27], although further experiments are required to support these conclusions. A small amount of tau has been found in dendrites as well, where its physiological role is not well understood yet but where it has been proposed to be involved in the regulation of synaptic plasticity [28]. Additionally, in the nucleus of neurons and non-neuronal cells, tau is believed to be key in maintaining the integrity of genomic DNA, cytoplasmic RNA and nuclear RNA [22,29]. Finally, other functions of tau include regulation of neuronal activity, neurogenesis, iron export and long-term depression (LTD) [30], although again, results are in some cases not conclusive due to discrepancies specially concerning the transgenic mouse lines used in the different experiments.

Interestingly, regulation of tau function is predominantly achieved through post-translational modifications, primarily phosphorylation at many sites. Thus, an increase in tau phosphorylation reduces its affinity for microtubules, resulting in neuronal cytoskeleton instability [31]. Hyperphosphorylation of tau may also induce pathology through other mechanisms. First, hyperphosphorylation of tau might induce tau missorting from axons to the somatodendritic compartment, which can cause synaptic dysfunction [32,33]. Second, degradation of tau (via autophagy or via proteasome) and its truncation by proteases can be also altered by phosphorylation. Third, often tau phosphorylation has been considered to enhance tau aggregation, as hyperphosphorylation and aggregation are both increased in AD [34]. However, this is still a matter of debate. Lastly, phosphorylation of tau may also change its interaction with other proteins. For example, the hyperphosphorylated form but not the unphosphorylated one can interact with the kinesin-associated protein JUN N-terminal kinase interacting protein 1 (JIP1) impairing the formation of the kinesin complex responsible of mediating axonal transport [35]. In the recent years, a novel post-translation modification of tau has been discovered. Acetylation of several lysine residues occurs by the action of the P300 acetyltransferase and the DNA cAMP response element (CREB)-binding protein. Depending on the sites, the acetylation of tau could inhibit its degradation, or by contrast, facilitate its degradation and suppress its phosphorylation and aggregation. [36,37]. Acetylation of tau has been found in AD and other taupathies. Specifically, acetylation at Lys174 has been recently identified in AD brains and it seems that it may contribute to retard tau turnover being critical for tau-induced toxicity, which has opened new therapeutic perspectives for the treatment of AD and other human tauopathies [38]. Finally, it is worth to note that tau protein is also subject to other post-translational modifications, including glycosylation, glycation, deamidation, isomerization, nitration, methylation, ubiquitylation, sumoylation and truncation [39]. Among them, *N*-glycosilation as well as the non-enzymatic modifications including deamidation, are believed to contribute to tau aggregation by changing its conformational structure and reducing its affinity for microtubules [40,41,42]. In contrast, other modifications such as the *O*-GlcNAcylation of tau (a type of *O*-glycosylation) may protect it against phosphorylation [43] and supress its aggregation [44]. Interestingly, in AD brains, *N*-glycosylation has been found to be increased [45] while *O*-GlcNAcylation seems to be reduced [43], which may explain the overall hyperphosphorylation and aggregation phenomena observed in the pathology. Other post-translational modifications, such as ubyquitylation and sumoylation, might influence tau degradation through the proteasome directly or indirectly, respectively [46,47]. Finally, tau truncation occurs in AD and other tauopathies and it is likely to play an important role, since tau fragments are not only prone to aggregation but they can also induce neurodegeneration independently of tau aggregation [30].

## 3. Tau Spreading Mechanisms

Although tau is predominantly an intracellular protein, recent evidence shows that, besides the amount of tau released by dying neurons, the protein can be also actively secreted to the extracellular space both in vitro and in vivo. In vitro, experiments have demonstrated on the one hand, that human tau can be secreted by non-neuronal and neuronal cell lines when overexpressed [48,49,50,51,52,53,54,55] and that this extracellular tau is toxic [56,57] and it may bind cellular receptors such as muscarinic (M1, M3) receptors [58]. In vivo, tau has been found in the interstitial fluid and the cerebrospinal fluid (CSF) of tau transgenic mouse brains before neurodegeneration, demonstrating that secretion is an active process not occurring only after cell death [59,60].

To date, mechanisms underlying tau spreading are not fully understood and further experiments are required to answer the main questions that are behind the process:

### 3.1. Which Are the Seeding Mechanisms?

The formation of tau filaments can be initiated or accelerated by the addition of seeds. Since 2009, several works have proved that Tau assemblies, when applied extracellularly, can seed the formation of aggregates [6,7] that serve as the start point for the spreading of tau. Considering that tau is mainly an intracellular protein, its propagation requires seeding as well as aggregate uptake and secretion. However, a lack of consensus exists regarding this point. In 2014, and thanks to super-resolution imaging techniques, Michel et al. demonstrated that monomeric tau could function as an efficient seed [61]. In contrast, the following year, Falcon et al. postulated that tau is only able to seed aggregation when it is competent, a characteristic that, according to their experiments, monomeric tau does not seem to show and that is absent when some amino acids are deleted from the full-length form [62]. Also in 2015, a work published by Mirbaha et al. supported the idea of bigger sizes of tau other than monomers to induce seeding, proposing tau trimers as the minimal particle size to be uptaken by a cell to serve as a conformational template for intracellular tau [63], an hypothesis that confirmed a previous work published in 2013 by Wu et al. [64]. More recently, a study by Fitzpatrick et al. has stressed the importance of the atomic structure characterization of tau filaments to understand their aggregation. Thus, the authors report how different protofilament packing interactions lead to ultrastructural polymorphism in tau filaments and more importantly, that some of those aggregation patterns seem to be common among AD patients [65].

Another important point is how mutations in tau protein can affect both its ability to seed or to be seeded. Thus, it has been demonstrated that the frequency of tau seeding appears to depend, in part, on the mutational status of the protein and the transduced fibrils [66,67,68]. The efficiency of mutated tau to induce seeding may also open the debate of tau propagation in tauopathies, where mutant tau is not a common feature in many cases. 

Tau isoforms also influence tau seeding, as it has been demonstrated by several groups in vitro when using different tau isoforms as seeding templates [69,70] and even by using purified oligomers isolated from brains of individuals with progressive supranuclear palsy (PSP) [71]. 

Aggregation may be directly influenced by the tau phosphorylation status. A recent publication has demonstrated how hyperphosphorylation is a driving force for tau aggregation in vitro, and how a specific phosphorylation pattern (Ser202/Thr205/Ser208) is sufficient to induce this phenomenon without the addition of any exogenous aggregation inducer [72].

Finally, tau strains seem to be essential for the overall propagation process including the seeding of tau. Thus, tau forms multiple unique prion-like strains with distinct biochemical properties that are able to induce diverse pathological phenotypes both in vitro and in vivo. These tau strains also target different brain regions and propagate pathology at unique rates [68,73].

### 3.2. Which Are the Releasing Mechanisms?

Although the precise mechanisms by which tau reaches the extracellular space remain to be elucidated, it is believed that its secretion occurs via the unconventional vesicular or non-vesicular-mediated secretory pathway, since tau protein does not contain an apparent signal sequence to regulate its translocation to the endoplasmic reticulum (ER) as it happens in the conventional secretory pathway [74]. One of the possible mechanisms within this unconventional secretory pathway is the vesicular-mediated exososome pathway. Exosomes are extracellular vesicles that are released upon fusion of the multivesicular bodies with the plasma membrane [75]. Several studies have demonstrated tau secretion via this pathway. For example, tau from the CSF [53] and from blood of patients with AD [76] was demonstrated to be associated with exososomes. Tau secretion in an exososome-dependent manner has been also reported in experiments with N2a cells overexpressing tau [77] as well as mediated by microglia cells [78]. Another vesicular-mediated mechanism proposed by Dujardin et al. in 2014 implicates ectosomes, which are larger extracellular vesicles that directly shed from cells by plasma membrane budding [79]. In the study, the authors postulate that tau is predominately secreted in ectosomes and when it accumulates, the exosomal pathway is activated [80]. A third mechanism proposes the formation of thin membranous bridges termed tunnelling nanotubes (TNTs) to mediate tau release and spreading [81]. These structures have been demonstrated to mediate neuron-to-neuron transfer of pathological tau protein assembles and therefore, they have been considered a possible highway in the spreading of tau, another prion-like protein in neurodegenerative diseases [82,83].

However, other studies report that the majority of extracellular tau is membrane-free [48,49] and that consequently, the extracellular vesicle-mediated mechanisms are only responsible for a small fraction of the total tau that is released to the extracellular space. Alternatives for tau secretion are not well understood yet and include the implication of some chaperone complexes [84] and certain Rab GTPases such as Rab7a [85] and Rab1a [86], supporting the idea that tau release involves intracellular vesicle transport.

Several types of stimuli affect tau secretion. For example, the stimulation of neuronal activity both in vitro and in vivo enhances tau release [87,88,89] and at the same time, extracellular tau influences tau activity [90], suggesting a positive feedback loop. On the other hand, lysosomal dysfunction or starvation also seems to increase tau secretion [91]. Finally, differences in tau species and isoforms, not only influence seeding as it was above-mentioned, but they also impact its release, as it has been reported for tau mutated forms [49], hyperphosphorylated forms of the protein [52,90] and truncated forms of tau [52].

### 3.3. Which Tau Species Are Secreted?

Elucidating which tau species are released to the extracellular space might help to understand the overall propagation process and it would be especially interesting for the development of therapies focused on halting tau spreading. As in the other key questions relative to the process, there is no consensus regarding tau species involved in secretion as to date, mainly because of the distinct nature of the experiments, making extrapolation to the actual physiological and/or pathological conditions difficult. Thus, while some cell lines such as M1C and Hela cells overexpressing human tau release a cleaved form of the protein in its C-terminal end [48,49,87], primary cortical neurons or neuronal cell lines such as SH-SY5Y secrete endogenous full-length tau [49,54]. The phosphorylation status of tau has been also examined. Depending on the cell type, secretion of overexpressed human tau by non-neuronal cell lines is either phosphorylated or importantly dephosphorylated at several sites [48,52]. 

Further experiments are required to determine which forms of tau could be preferentially released in pathological situations in vivo and whether some forms are associated with particular stimuli.

### 3.4. Which Are the Uptaking Mechanisms?

Initially, a role for “bulk” or “fluid phase” endocytosis was proposed, but the precise mechanism was unknown. In a study published in 2013, Holmes et al. demonstrated the uptake of tau and α-synuclein aggregate seeds by neurons through macropinocytosis, a subtype of fluid-phase bulk endocytosis, which might be the most likely mechanism for tau uptake. This process is initiated by the binding of aggregated protein on the cell surface to heparin sulphate proteoglycans (HSPGs), a family of core proteins decorated with glycosaminoglycan polysaccharides. Remarkably, the internalization process is only triggered by these aggregated species and not by monomeric tau [92]. Another study also suggests that HSPGs can mediate the internalization of exososomes [93]. Interestingly, this HSPGs-mediated process is similar to the one utilized by eukaryotic cells for virus internalization [94,95]. 

Not only neurons participate in the uptake of tau aggregates. For example, microglial cells are able to internalize and degrade pathological tau, a process that is enhanced by the use of a tau monoclonal antibody [96]. However, it is unknown whether cell-specific mechanisms exist.

### 3.5. How Does Propagation Occur?

Growing evidence suggests that accumulation of abnormal tau is mediated through spreading of protein seeds from cell to cell and involving extracellular tau species as the main agent in the interneuronal propagation of neurofibrillary lesions and spreading of tau toxicity throughout different brain regions [97,98]. As it has been previously described, pathogenic tau needs to be released from the originating neuron or glial cell and taken up by a neighbouring neuron or glial cell. Notably, in neurodegenerative diseases this transfer may occur across synapses and thus correspond to intrinsic connectivities between cells [99]. Importantly, some studies strongly suggest that tau can be spread via neuronal connections. Indeed, in experiments with animal models in which initial focus of the pathology is varied, it has been shown that different neuronal populations are sequentially affected depending on the injection site of the preformed fibrils [7,100,101], supporting the idea of neuronal connectivity as an important factor to understand cell vulnerability to protein aggregation in neurodegenerative diseases. This idea is further supported by the fact that structures that are anatomically distant are affected in some of these pathologies, indicating that transmission might not simply occur by simple diffusion among neighbouring cells [7,100,102]. 

Despite tau propagation being broadly reported in animal models of tauopathies, does it also occur in humans? If so, how does it happen? Neuropathological studies have identified that, in neurodegenerative diseases, stereotypical patterns of pathology take place over time, with the progression of these patterns being associated with the increasing severity of the clinical phenotype [103]. This was firstly established in AD patient brains, where tau pathology was found to propagate from the entorhinal cortex through the hippocampus and into the limbic and associated cortexes, which correlates with the clinical cognitive status of the patient [104]. Stereotypical patterns have been also found in other tauopathies such as chronic traumatic encephalopathy (CTE), a tauopathy that occurs as a consequence of repetitive mild traumatic brain injury, where tau lesions can originate closer to perivascular spaces within the depths of cortical sulci [105] and become subsequently detectable in larger regions of the neocortex and allocortex, diencephalon, basal ganglia, brainstem and spinal cord [106]. Alternative to the hypothesis of the spread of the pathology, the concept of neuronal vulnerability was introduced to explain the appearance of the pathologies in different brain regions [8]. This concept suggests that some neurons are intrinsically more vulnerable than others during pathogenic processes [107]. However, the factors that determine this “intrinsic vulnerability” are poorly understood and might include the expression of certain gene profiles that could make a neuron more dysfunctional at an earlier stage and structurally abnormal than others. 

Finally, it is noteworthy to stress the putative role of glia in the propagation of tau pathology. At least in animal models, the injection of brain lysates from different taupathies including progressive supranuclear palsy (PSP), corticobasal degeneration and argyripilic grain disease into the brains of mice expressing wild-type tau, induces tau pathology in oligodendrocytes and astrocytes [108]. This demonstrates the spreading to these cells, but does not provide an explanation about the potential role of glia in tau propagation. Interestingly, recently, work published by Venegas et al. has established a direct correlation between the microglia-dependent inflammasome activity and the spreading of amyloid-β pathology in AD [109]. Taking into account these results, it would be very informative to perform experiments to determine a possibly similar role for microglia and the inflammasome in the spreading of tau pathology.

Despite contribution of glial cells to the initiation and progression of several neurodegenerative disorders apart from tauophaties, further studies are required to elucidate the specific role of these brain cells in protein propagation.

## 4. Tau Spreading Implications for Dysfunctional Tauopathies

To date, we have no discovered effective therapies for the treatment of tauopathies, since neuroprotective and anti-inflammatory therapies have largely proved to be unsatisfactory. The discovery of tau spreading in neurodegenerative disorders has opened new perspectives for therapeutic strategies, focused on preventing or even stopping the process. 

As it was extensively discussed in this review, tau spreading involves several different processes from protein seeding and aggregate release, to the uptake of the extracellular tau and the final propagation of the toxic species. Consequently, therapies can be directed to prevent any or several parts of the process. For example, stabilization of the wild-type protein in its normal conformation conferring resistance to template-directed conformational change without interfering with its normal function could be a possible therapeutic approach [103] that indeed, has been already applied with success in the treatment of a neurodegenerative disorder, transthyretin amyloidosis [110]. Post-translational modifications, especially hyperphosphorylation, have been also the focus of many therapies. Indeed, some compounds are currently undergoing clinical trials, such as a derivative of methylene blue (LMTX), which has numerous targets including some tau-phosphorylating kinases, and that conferred treatment benefits in a Phase II trial in individuals with mild-to-moderate AD and now is undergoing a Phase III trial [111], or tideglusib, a GSK-3 inhibitor (the main tau kinase. [112]), which reduced the progression of brain atrophy in a Phase II trial in patients with mild-to-moderate PSP [113].

Another strategy could be addressed to interfere with the release or the uptake of the protein. Thus, specific antibodies could capture protein seeds in the extracellular space before being transferred to the receiving cell [114] or target receptors or other cellular proteins needed for the uptake or release of pathogenic proteins [115,116,117]. Immunotherapy has emerged as a promising therapeutic strategy for tauopathies. Although mechanisms of action remain unclear, experiments in transgenic mouse models with vaccinations have reduced tau pathology and improved the performance in behavioural tests [118,119]. It has been demonstrated that when recycling of antibodies between the central nervous system (CNS) and the plasma compartments exists, there is a reasonably high CNS exposure to the antibody [120]. Antibodies may enter neurons by different mechanisms [30] inhibiting intracellular tau aggregation, but they could also exert their therapeutic effects by interacting with extracellular antigens without entering cells [121]. Indeed, some antibodies were able to prevent tau spreading in transgenic mice by capturing extracellular tau seeds [122]; a promising result that could be relevant for its translational application in humans in the future. 

An important obstacle when translating these therapies to humans will be the blood–brain barrier (BBB) and the blood–cerebrospinal fluid barrier, both of which limit the passage of extrathecally administered antibodies into the CNS [123]. In this way, it has been recently suggested that the use of sound waves could enhance BBB permeability, thereby facilitating the delivery of tau antibodies to the brain [124]. It is worth mentioning that some compounds used in clinical practice for decades have been demonstrated to pass freely through the BBB and to be effective in the treatment of some neurodegenerative disorders. This is the case for ceftriaxone, a β-lactam antibiotic used for halting or reversing neurodegeneration in some disorders such as Alexander’s Disease and Parkinson's Disease, thanks to its chaperon-like effect on some of the misfolded proteins involved in these pathologies (GFAP and α-synuclein, respectively) [125,126,127].

On the other hand, interfering with cellular uptake or release would probably affect the homeostasis of other cellular proteins leading to important adverse effects, and consequently, it should be taken into account when designing a therapeutic strategy.

## 5. Conclusions

The existence and subsequent relevance of tau spreading in tauopathies has been relatively recently highlighted. Although growing evidence has emerged supporting and demonstrating this phenomenon, mechanisms remain poorly understood and further studies are required to understand the process and its consequences. Hence, tau spreading has been the focuse of many therapeutic strategies, which to date, have collected promising results. However, a detailed knowledge of how the spreading may occur is fundamental for the success of these therapies. Consequently, intensive work is required so that the propagation of tau pathology is considered as a real target for future therapeutic approaches.

## Figures and Tables

**Table 1:** Classification of tauopathies.

Tauopathies	Tau 3R/4R Ratio
**Primary Tauopathies**	
Richardson’s syndrome	1:2–4
Pick’s disease	3:1
Frontotemporal dementia with parkinsonism-17 (FTDP-17)	1:2
Postencephalic parkinsonism (PEP)	1:1
Argylophilic grain disease	1:2
Corticobasal degeneration	1:2
Progressive supranuclear palsy (PSP)	1:3–4
Parkinson-dementia complex (PDC Guam)	1:1
Guadeloupean parkinsonism	1:2
**Secondary Tauopathies**	
Alzheimer’s Disease (AD)	1:1
Creutzfeldt–Jakob disease	-
Down’s syndrome	1:1
Dementia pugilistica	1:1
Familial British Dementia	-
